# Expression of gasdermin D in clear cell renal cell carcinoma and its effect on its biological function

**DOI:** 10.3389/fonc.2023.1163714

**Published:** 2023-07-06

**Authors:** Jichi Zhang, Yujie Wang, Jun Ma, Ainiwaer Aimudula

**Affiliations:** ^1^ Urological Center, Xinjiang Medical University, The First Affiliated Hospital of Xinjiang Medical University, Urumqi, China; ^2^ Cancer Center, Xinjiang Medical University, The First Affiliated Hospital of Xinjiang Medical University, Urumqi, China

**Keywords:** ccRCC, GSDMD, bioinformatics, apoptosis, transwell, MTT, immunohistochemistry

## Abstract

**Background:**

Clear cell renal cell carcinoma (ccRCC) is the most common type of renal cell carcinoma, which suffers from the lack of diagnosis and treatment methods, and many patients cannot be diagnosed at first time. Gasdermin D (GSDMD) is involved in inflammatory reactions and pyroptosis and is considered a potential therapeutic target. This paper’s aim is to elucidate the expression of GSDMD in clear cell renal cell carcinoma and its value for treatment and prognosis, as well as its impact on the biological function of clear cell renal cell carcinoma.

**Method:**

The Cancer Genome Atlas (TCGA) database was used to compare the expression of GSDMD in tumor and normal tissues, analyze its correlation with cancer stage and overall survival time, and establish receiver operating characteristic (ROC) curve, which was confirmed by the Gene Expression Omnibus (GEO) database and immunohistochemical staining of clinical samples and PCR and Western blotting (WB) of cell lines. The relationship between GSDMD and patient prognosis and staging was analyzed using TCGA database and validated using clinical sample data. Differentially expressed genes (DEGs) and epithelial–mesenchymal transition (EMT)-related genes of GSDMD were screened by TCGA database. Protein–protein interaction (PPI) of GSDMD was constructed by GeneMANIA and STRING, and Gene Ontology (GO) and Kyoto Encyclopedia of Genes and Genomes (KEGG) enrichment were analyzed by the Metascape database. Then, R software was used to analyze the immune cell infiltration, immune microenvironment score, and tumor mutational burden (TMB) analysis of GSDMD high- and low-expression groups in TCGA database. GSDMD lentivirus was used to transfect 769-P cells to construct stable upregulated and downregulated transfected cell lines. PCR was used to verify the expression differences of differentially expressed genes between the high- and low-expression groups of GSDMD; then, MTT, flow apoptosis, and Transwell were used to detect the proliferation, apoptosis, invasion, and migration of the transfected cells.

**Results:**

The results of bioinformatics analysis showed that the expression of GSDMD in clear cell renal cell carcinoma was significantly correlated with patient stage and overall survival, and the tumor with high expression of GSDMD had a worse stage and overall survival. GSDMD has some significance in the diagnosis of ccRCC. The results of EMT correlation analysis and enrichment analysis showed that GSDMD was correlated with genes and pathways related to invasion and metastasis of renal cell carcinoma. The subsequent immune cell infiltration analysis showed that there were many differences in the infiltration of immune cells between the high- and low-expression groups of GSDMD, such as naive B cells. The immune microenvironment score showed that the high-expression group had a lower proportion of stromal cells than the local expression group but had a higher proportion of immune cells. Through TMB, it was shown that the high-expression group had a higher mutation. The expression of GSDMD in renal cell carcinoma by immunohistochemistry and *in vitro* cell experiments was confirmed. According to the prognostic information of clinical patients, it was found that GSDMD was significantly correlated with TNM stage, Fuhrman grade, lymph node metastasis, gender, and smoking or not, and the prognosis of patients with high expression of GSDMD was worse. After that, we constructed stable transfection cell lines with high expression and knockdown through lentivirus transfection and verified the expression amount of differentially expressed genes by PCR, which is consistent with the results of TCGA database. Then, we confirmed that GSDMD is related to proliferation, invasion, migration, and apoptosis of ccRCC by MTT, flow apoptosis, and Transwell assay. The low expression of GSDMD inhibits the proliferation, invasion, and migration of tumors and enhances apoptosis and vice versa. Therefore, GSDMD can be used as a potential biological marker for the diagnosis and prognosis of ccRCC.

## 1 Introduction

Clear cell renal cell carcinoma (ccRCC) is a common tumor of the urinary system, accounting for approximately 80% of renal cell carcinoma (RCC) (1). The early treatment of ccRCC is mainly surgical treatment, but approximately 17% of patients have distant metastasis (2, 3) at the time of definite diagnosis. Moreover, due to the high variability of clear cell renal cell carcinoma and the lack of definite diagnostic and therapeutic markers (4), the difficulties of early diagnosis and subsequent treatment are aggravated. Therefore, finding new and effective prognostic biomarkers can improve the therapeutic efficacy and long-term prognosis of ccRCC patients.

Gasdermin D (GSDMD) belongs to the gasdermin (GSDM) protein family, which includes GSDMA, GSDMB, GSDMC, GSDMD, GSDME (DFNA5), and PJVK (DFNB59). GSDMD plays an important role in inflammation and pyroptosis, and GSDMD is the most important effector cell of pyroptosis. At present, many studies have shown that GSDMD plays an important role in the occurrence and development of various cancers (5–9).

However, the role of GSDMD in the occurrence and development of ccRCC has not been clearly confirmed by experiments. The expression of GSDMD in clear cell renal cell carcinoma and its effect on its biological function was investigated by means of a bioinformatics platform database, clinical data analysis, and *in vitro* cytological experiments.

## 2 Materials and methods

### 2.1 Tissue specimen preparation

Sixty-five patients who received renal cell carcinoma surgery from December 2014 to December 2019 in the First Affiliated Hospital of Xinjiang Medical University were randomly selected. All specimens were confirmed as ccRCC by the Department of Pathology of Xinjiang Medical University. All patients did not receive any anti-tumor treatment such as radiotherapy and chemotherapy before the operation. This research obtained approval from the Medical Ethics Committee of the First Affiliated Hospital of Xinjiang Medical University (K202212-06). 786-O, 769-P, and HK-2 cell lines were purchased from Procell Life Science and Technology Co., Ltd.

### 2.2 Data collection related to expression and prognosis of renal cell carcinoma

The related data were collected from RNA-seq data in level 3 HTSeq-FPKM format of KIRC project in The Cancer Genome Atlas (TCGA) database. Subsequently, RNA-seq data in FPKM (Fragments Per Kilobase per Million) format was converted into TPM (transcripts per million reads) format, and log2 was converted (at the same time, the related data of survival, prognosis, and staging were obtained). After that, the differential expression of 539 cancer tissues and 72 normal kidney tissues was analyzed by the Wilcoxon rank sum test. According to the median expression of GSDMD, 539 cancer tissues were divided into the high-expression group and the low-expression group. The Kaplan–Meier (K-M) method was used to draw the survival curve, and the log-rank test was used to calculate the statistical significance. The R language Survival ROC package was used to draw the receiver operating characteristic (ROC) survival curve.

The dataset of the Gene Expression Omnibus (GEO) database GSE53757 included 144 samples, 72 cancer samples, and 72 normal samples. The R package “ggpubr” was used to draw a boxplot of the intergroup differences of GSDMD in normal and tumor samples of clear cell renal cell carcinoma, and wilcox was used to calculate the significance between groups. The R package “ROCR” was used to draw the ROC diagnostic curve of GSDMD in the GSE53757 dataset.

### 2.3 GSDMD differentially expressed genes and epithelial–mesenchymal transition-related genes were collected

Relevant data were collected from RNA-seq data in level 3 HTSeq-Counts format in TCGA (https://portal.gdc.cancer.gov/) KIRC project.

### 2.4 Construction of protein–protein interaction network

GeneMANIA (http://genemania.org) database and STRING (https://string-db.org/) database were used to construct protein–protein interaction (PPI) to find proteins and genes interacting with GSDMD. In the STRING database, the first 10 genes interacting with GSDMD were selected after species selected humans. Based on the GeneMANIA database, human was selected, GSDMD was input, and the protein cooperation network after selection and operation was achieved.

### 2.5 GO and KEGG analyses

The Metascape (https://metascape.org) database was used to analyze Gene Ontology (GO) and Kyoto Encyclopedia of Genes and Genomes (KEGG) enrichment of differentially expressed genes and interacting genes of GSDMD.

### 2.6 Immune cell infiltration, immune microenvironment score, and tumor mutational burden analysis

CIBERSORT is a method published in *Nature Methods* in 2015 and widely used in immune cell analysis. CIBERSORT is a deconvolution algorithm developed by Binder G et al., which can calculate the cell composition of complex tissues based on standardized gene expression data. This method can quantify the abundance of specific cell types. CIBERSORT can quantify cell components from input gene expression profile data. The CIBERSORT package was used to perform immune infiltration analysis on samples between the high- and low-expression groups of GSDMD in TCGA-KIRC dataset. Samples were screened based on p-values <0.05, and Wilcox was used to compare the infiltration differences of significantly infiltrating immune cells between the high- and low-expression groups. Spearman’s and Mantel’s tests were further used to calculate the correlation between GSDMD gene and immune cells.

The “estimate” package was used to perform an estimated analysis on the high- and low-expression groups of GSDMD in TCGA-KIRC dataset, and the “ggplot2” package was used to draw violin diagrams using the wilcox.test test method. Spearman’s and Mantel’s tests were further used to calculate the correlation between GSDMD gene and immune microenvironment score.

We calculated the tumor mutational burden (TMB) values of each sample based on the mutation data of clear cell renal cell carcinoma in TCGA database (calculation formula: number of mutations/length of detected exon Mb), tested the TMB differences between the high- and low-expression groups of GSDMD using wilcox.test, and calculated Spearman’s correlation between GSDMD and TMB.

### 2.7 Cell culture and lentivirus transfection

769-P cells were cultured in Dulbecco’s modified Eagle’s medium (DMEM) high glucose medium containing 10% fetal bovine serum and 1% penicillin and streptomycin and placed in a constant temperature incubator with 5% CO_2_ and 37°C. LV, LV-NC, si, and si-NC were transfected into 769-P cells according to the operation manual provided by Jikai Company, and puromycin was used to screen stable and high-expression GSDMD transfected cell lines LV and LV-NC and stable knockdown GSDMD transfected cell lines si and si-NC. The transfection efficiency was verified using PCR and used for subsequent experiments.

### 2.8 Western blotting

The total protein was extracted by radioimmunoprecipitation assay (RIPA) lysate, and the protein concentration was determined by bicinchoninic acid (BCA) protein quantitative kit. The sodium dodecyl sulfate–polyacrylamide gel electrophoresis (SDS-PAGE) gel was prepared, 40 μg of total protein was taken for electrophoresis, and then the membrane was transferred and sealed with a rapid sealing solution for 15 min. GSDMD (1:1,000) and GAPDH (1:1,000) were added to the primary antibody and incubated overnight in a shaking table at 4°C. The next day, the second antibody was added and incubated at room temperature for 2 h; the film was washed with TBST, exposed to enhanced chemiluminescence (ECL) developer, and photographed by a gel imager; the gray value of each band was measured by ImageJ software.

### 2.9 RT-qPCR

The total RNA was extracted by RNA extraction kit, and 500 ng of total RNA was transcribed into cDNA by RT Easy™ II kit. Finally, the CT values of GSDMD and GAPDH were detected by Real Time PCR Easy™-SYBR Green I kit, and the relative expression of GSDMD was calculated by 2^−ΔΔCt^. The reaction conditions of PCR were as follows: 95°C for 3 min, 95°C for 10 s, and 58°C for 30 s for 40 cycles. GSDMD sequence is as follows: GSDMD: F 5′-CTCTGCCCTCCTTCGAGCAC-3′ GSDMD R 5′-CTGCAGCCACAAATAACTCAGCTT-3′; GAPDH F 5′-AGAAGGCTGGGGCTCATTTG-3′ GAPDH R 5′-AGGGGCCATCCACAGTCTTC-3′.

### 2.10 Immunohistochemistry, evaluation, and follow-up

#### 2.10.1 Immunohistochemistry and evaluation

After immunohistochemical staining, images were collected and data were recorded. The results of GSDMD staining were analyzed by IPP 6.0 software. The average optical density (AOD) was calculated as AOD = IOD/Area; the value was between 0 and 1, and the AOD value represents the staining intensity.

#### 2.10.2 Follow-up

Sixty-five patients who received renal cell carcinoma surgery from December 2014 to December 2019 in the First Affiliated Hospital of Xinjiang Medical University were selected, and related clinicopathological data such as gender, age, clinical stage, pathological grade, operation time, diabetes history, and smoking history were collected. The follow-up time ranged from 10 to 95 months, with a median time of 70 months and January 2023 as the follow-up deadline. The time from the operation day to the patient’s death due to disease and the time of first recurrence and metastasis were recorded.

#### 2.10.3 MTT test

The proliferation ability of transfected 769-P cells was detected by MTT assay. After trypsin digestion, the transfected 769-P cells were counted on a counting plate and inoculated into a 96-well plate at 5 × 10^3^ cells per well. After 1, 2, 3, 4, 5, 6, and 7 days of culture, 20 μl of MTT reagent was added to each well and incubated at 37°C for 4 h, the 96-well plate was taken out, the liquid was sucked out, and 150 μl of dimethyl sulfoxide (DMSO) was added to each well. The OD value of 490-nm wavelength was detected after oscillation in the microplate reader for 200 s.

#### 2.10.4 Apoptosis test

The cells and their supernatants were collected by trypsin without EDTA, washed twice with phosphate-buffered saline (PBS), diluted with 10× buffer to 1×, and then transferred to 1.5-ml EP tube by blowing and mixing with 100 μl of cell precipitates. Annexin V and 7-ADD measuring 5 μl were added into 100-μl cell suspension, mixed evenly, and incubated at room temperature for 15 min; then, 400 μl of buffer was added into an EP tube, and fluorescence detection was carried out by flow cytometry.

#### 2.10.5 Transwell invasion and migration experiment

Matrigel measuring 60 μl (or 50 μg/chamber) was added to 300 μl of serum-free medium and mixed well, and 100 μl was added to the upper chamber and placed into an incubator at 37°C for 4–5 h. The cells were digested by trypsin and counted. The cell suspension with a concentration of 5 × 10^4^ was prepared using the serum-free medium. Matrigel was washed with serum-free medium once, and then 200 μl of cell suspension was added to each upper chamber; 700 μl of culture medium containing 20% fetal bovine serum (FBS) was added into the lower chamber and incubated in a 37°C incubator for 24 h. After that, it was taken out of the Transwell chamber and washed with PBS twice, fixed with 4% paraformaldehyde at room temperature for 15 min, and then added into 0.1% crystal violet solution for dyeing at room temperature for 30 min. After being cleaned with PBS twice, the matrix glue and cell dye on the upper ventricle side were wiped off and observed, and pictures were taken under an inverted microscope. The cells were counted using IPP (the steps of the migration experiment and invasion experiment are similar, so we will not repeat them here).

### 2.11 Statistical analyses

The experimental data were processed by SPSS 26.0. Mean ± standard deviation (x̄ ± SD) was used to express the measurement data. If the data conform to the normal distribution and meet the homogeneity of variance, a t-test was used to compare the two groups, and one-way ANOVA was used to compare among multiple groups. Classified data were analyzed by chi-square test. Wilcoxon rank-sum test was used for quantitative data and ordered classification variables that did not conform to normal distribution. The survival curves were drawn by the Kaplan–Meier method and compared by the log-rank test. p < 0.05 was considered statistically significant.

## 3 Results

### 3.1 Expression of GSDMD in ccRCC

**Figure 1:**
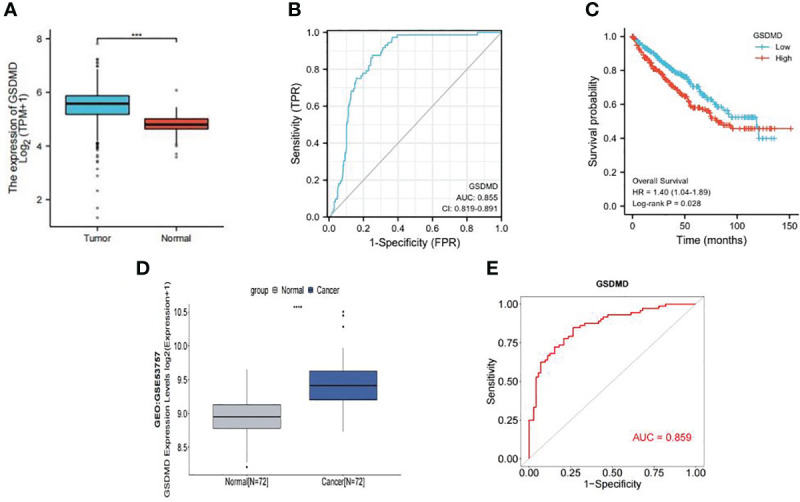
Expression of GSDMD in tumor and its prognostic value. **(A)** Expression of GSDMD in tumor tissues (n = 539) and normal tissues (n = 72) in TCGA database (p < 0.0001). **(B)** The ROC curve of tumor tissues (n = 539) and normal tissues (n = 72) in TCGA database (AUC = 0.855). **(C)** Kaplan–Meier survival plots of overall survival in ccRCC. Red line represents higher expression of GSDMD, and blue one lower expression. X-axis means survival time (month). Y-axis means survival probability. The log-rank test indicates that p = 0.028 and HR = 1.40 (1.04–1.89), which means that GSDMD is significantly associated with the prognosis of ccRCC. **(D)** Expression of GSDMD in tumor tissues (n = 72) and normal tissues (n = 72) in GEO database (p < 0.0001). **(E)** The ROC curve of tumor tissues (n = 72) and normal tissues (n = 72) in TCGA database (AUC = 0.859). GSDMD, gasdermin D; TCGA, The Cancer Genome Atlas; ROC, receiver operating characteristic; AUC, area under the curve; ccRCC, clear cell renal cell carcinoma; HR, hazard ratio; GEO, Gene Expression Omnibus.

We compared the expression of GSDMD in 539 cases of clear cell renal cell carcinoma and 72 cases of normal kidney tissue through TCGA database screening. The results showed that the expression of GSDMD in clear cell renal cell carcinoma was significantly higher than that in normal renal tissue (**Figure 1A**). Then, according to the information of 539 patients with ccRCC and 72 normal patients in TCGA database, we made the ROC curve (**Figure 1B**), and the results showed that the area under the curve (AUC) = 0.855, which showed that GSDMD has a good diagnostic significance for ccRCC and normal people, indicating that GSDMD is a potential diagnostic index for ccRCC. Finally, according to the median expression of GSDMD, we divided the patients into the high-expression group and low-expression group and drew the K-M curve (**Figure 1C**). It can be seen that there is a significant difference in the overall survival (OS) time between the high-expression group and the low-expression group, suggesting that the expression of GSDMD is related to the prognosis of tumor patients. Subsequently, we validated TCGA data using the expression data of GSDMD in the GEO database. The results showed that there was a significant statistical difference (p < 0.001) between the two groups of specimens in the GSE53757 dataset of 72 normal and 72 tumor specimens (**Figure 1D**), and the ROC curve plotted based on the patient’s diagnosis and expression information also showed an AUC value of 0.859 (**Figure 1E**), indicating that the expression level of GSDMD has good significance for the classification and diagnosis of clear cell renal cell carcinoma. This also verifies the information in TCGA database.

### 3.2 Analysis of the correlation between GSDMD and stage in ccRCC

According to the data of ccRCC provided by TCGA database, we compared the difference in clinical indexes between the high-expression group and low-expression group of GSDMD. As shown in **Table 1**, it was statistically significant in the clinical stage and pathological stage of ccRCC, and its stage increased correspondingly with higher expression of GSDMD.

**Table 1:** Comparison of GSDMD expression and tumor stage.

Characteristic	Overall	Low expression of GSDMD	High expression of GSDMD	p
n	539	269	270	
T stage, n (%)				0.004
T1	278 (51.6%)	158 (29.3%)	120 (22.3%)	
T2	71 (13.2%)	25 (4.6%)	46 (8.5%)	
T3	179 (33.2%)	80 (14.8%)	99 (18.4%)	
T4	11 (2%)	6 (1.1%)	5 (0.9%)	
Pathologic stage, n (%)				0.002
Stage I	272 (50.7%)	157 (29.3%)	115 (21.5%)	
Stage II	59 (11%)	22 (4.1%)	37 (6.9%)	
Stage III	123 (22.9%)	57 (10.6%)	66 (12.3%)	
Stage IV	82 (15.3%)	32 (6%)	50 (9.3%)	

The difference in GSDMD expression is statistically different from the tumor T stage (p = 0.004) and the pathologic stage (0.002).

GSDMD, gasdermin D.

### 3.3 DEGs in ccRCC

**Figure 2:**
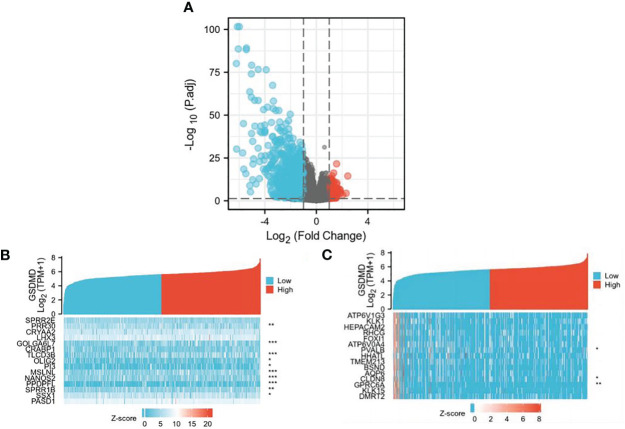
Differential expression of GSDMD gene among different expression groups in ccRCC. **(A)** The volcano plot of differentially expressed genes (DEGs). After adjusting p-value <0.05 and |Log2-FC| > 1, the blue dot represents the downregulated genes (n = 421), and the red dot represents the upregulated genes (n = 86). **(B)** Heatmap of correlation between the first 15 upregulated genes and GSDMD. **(C)** Heatmap of correlation between the first 15 downregulated genes and GSDMD. GSDMD, gasdermin D; ccRCC, clear cell renal cell carcinoma. *(p<0.05), **(p<0.01), ***(p<0.001).

We found 507 differentially expressed genes between the high-expression group and the low-expression group of GSDMD (after adjusting p-value <0.05 and |Log2-FC| > 1), among which 86 differentially expressed genes were highly expressed (accounting for 17.0%) and 421 differentially expressed genes were expressed at low levels (accounting for 83.0%) (**Figure 2A**). Among the first 15 upregulated genes, the ones with a statistical difference in correlation with GSDMD were PRR30, GOLGA6L7, TLCD3B, OLIG2, PI3, MSLNL, NANOS2, PPDPFL, SPRR1B, and SSX1 (**Figure 2B**). Among the first 15 downregulated genes, the ones with the statistical difference in correlation with GSDMD were PVALB, CLDN8, and GPRC6A (**Figure 2C**).

### 3.4 GO and KEGG enrichment analyses of DEGs

**Figure 3:**
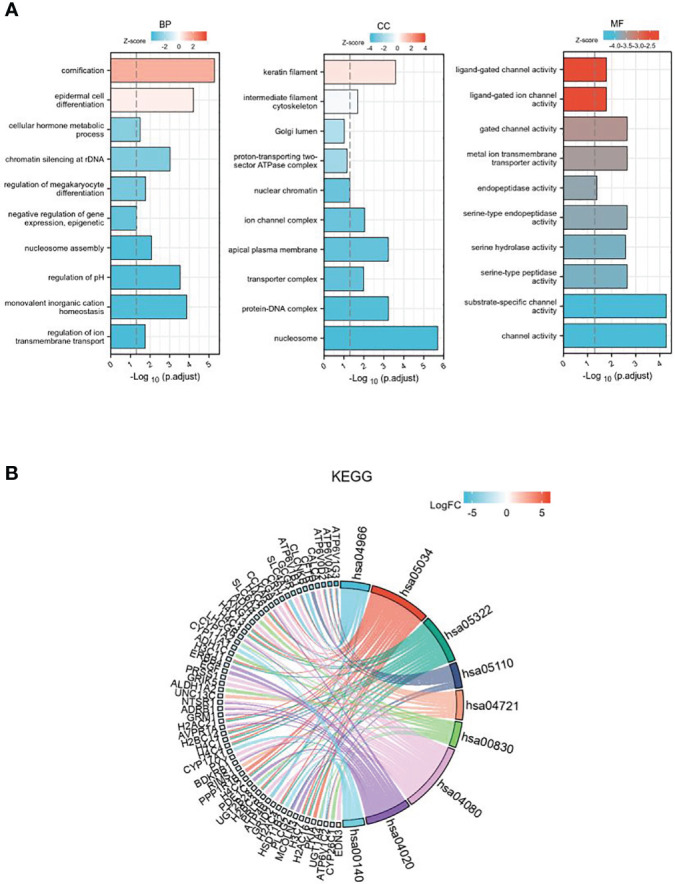
The GO and KEGG enrichment analysis of DEGs. **(A)** GO enrichment in biological processes, cellular components, and molecular function for the 507 DEGs. **(B)** KEGG pathway enrichment analysis of the 507 DEGs. GO, Gene Ontology; KEGG, Kyoto Encyclopedia of Genes and Genomes; DEGs, differentially expressed genes.

GO enrichment analysis includes three parts: biological process (BP), cell components (CC), and molecular function (MF) (**Figure 3A**). The results show that differentially expressed genes related to GSDMD are mainly enriched in the nucleosome, ion channel complex, transporter complex, apical plasma membrane, keratin filament, etc. The main functions include cornification, regulation of pH, chromatin silencing at rDNA, regulation of megakaryocyte differentiation, regulation of ion transmembrane transport, cellular hormone metabolic process, negative regulation of gene expression, and epigenetics. In addition, KEGG pathway enrichment analysis shows (**Figure 3B**) that these differential genes are mainly enriched in Collecting duct acid secretion, Alcoholism, Synaptic vesicle cycle, Retinol metabolism, Calcium signaling pathway, Steroid hormone biosynthesis, and other pathways.

### 3.5 Establishment of GSDMD PPI and analysis of its functional enrichment

**Figure 4:**
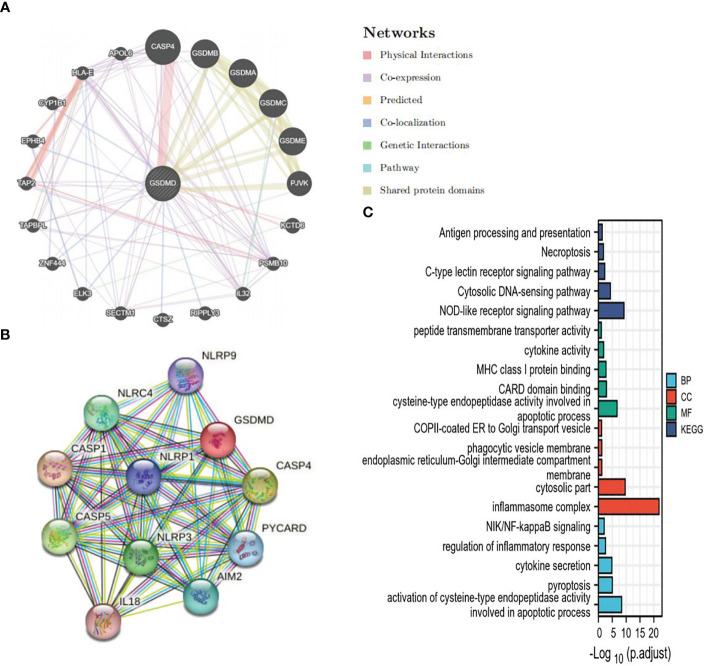
GSDMD PPI and analysis of its functional enrichment. **(A)** The PPI network of GSDMD interaction partners is generated by the GeneMANIA database. **(B)** The PPI network of GSDMD interaction partners is generated by the STRING database. **(C)** GO and KEGG enrichment analysis of interaction partners. GSDMD, gasdermin D; PPI, protein–protein interaction; GO, Gene Ontology; KEGG, Kyoto Encyclopedia of Genes and Genomes.

In order to further study the mechanism of action of GSDMD, we constructed a PPI of GSDMD through STRING (**Figure 4A**) and GeneMANIA (**Figure 4B**) and screened out 24 genes in addition to GSDM family, including NLRP9, NLRC4, NLRP1, NLRP3, CASP1, CASP4, CASP5, PYCARD, IL18, AIM2, KCTD6, PSMB10, IL32, RIPPLY3, CTSZ, SECTM1, ELK3, ZNF444, TAPBPL, TAP2, EPHB4, CYP1B1, HLA-E, and APOL6. GO and KEGG enrichment analyses of these genes were performed again. GO enrichment analysis showed that these interaction factors were mainly located at the locations of the inflammasome complex, cytosolic part, phagocytic vesicle membrane, etc., and played a role in the apoptotic process, cytokine activity, peptide transmembrane transporter activity, NOD-like receptor signaling pathway, Necroptosis, Antigen processing and presentation, Cytosolic DNA-sensing pathway, NIK/NF-kappaB signaling, regulation of inflammatory response, pyroptosis, cytokine secretion, and other processes and pathways (**Figure 4C**).

### 3.6 Correlation between GSDMD and expression of epithelial–mesenchymal transition-related molecules

**Figure 5:**
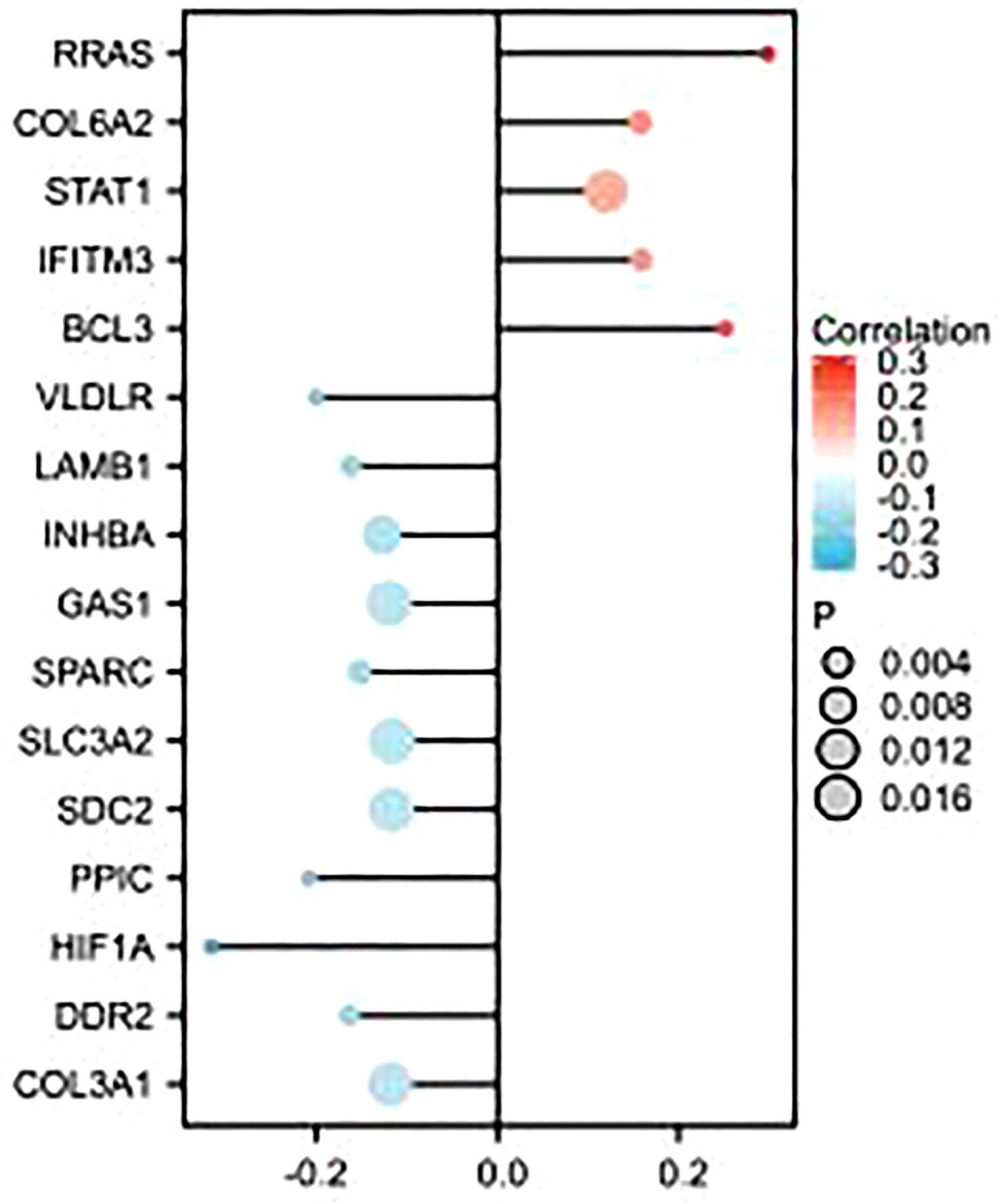
Correlation analysis between GSDMD- and EMT-related molecules. This figure represents the correlation analysis between GSDMD- and 16 EMT-related molecules. GSDMD is positively correlated with BCL3, IFITM3, STAT1, COL6A2, and RRAS and has statistical significance in KIRC. GSDMD is negatively correlated with COL3A1, DDR2, HIF1A, PPIC, SDC2, SLC3A2, SPARC, GAS1, INHBA, LAMB1, and VLDLR and has statistical significance in KIRC. GSDMD, gasdermin D; EMT, epithelial–mesenchymal transition.

As shown in the figure, epithelial–mesenchymal transition (EMT)-related molecules have a strong correlation with GSDMD in many tumors, as well as in ccRCC, where GSDMD is positively correlated with BCL3, IFITM3, STAT1, COL6A2, and RRAS and significantly negatively correlated with COL3A1, DDR2, HIF1A, PPIC, SDC2, SLC3A2, SPARC, GAS1, INHBA, LAMB1, and VLDLR (**Figure 5**).

### 3.7 Immune cell infiltration, immune microenvironment score, and tumor mutational burden analysis

**Figure 6:**
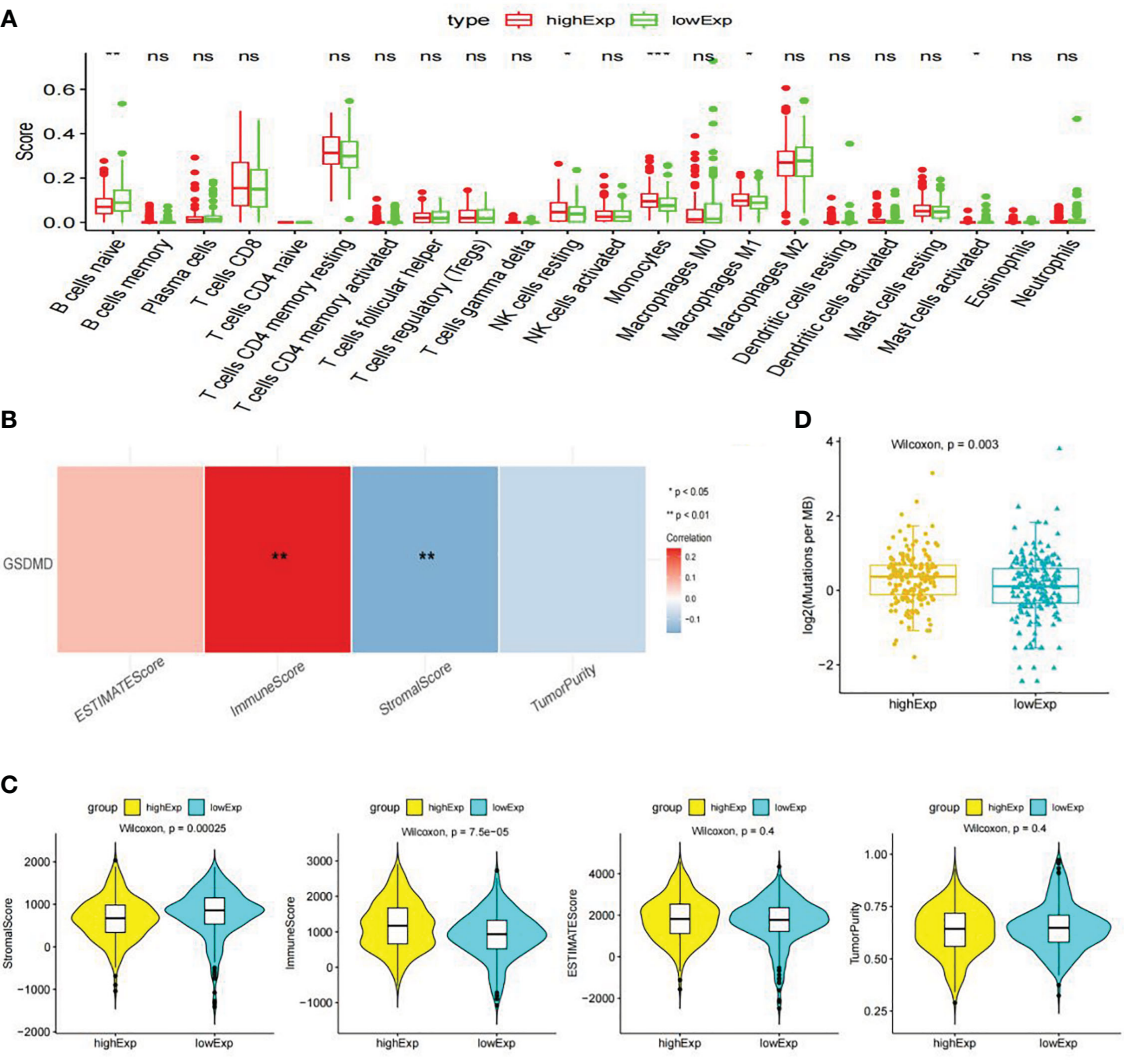
Immune cell infiltration, immune microenvironment score, and tumor mutational burden analysis. **(A)** Display information on immune cell infiltration. **(B)** The following graph shows Spearman’s correlation heatmap between GSDMD expression and significant differences in immune cells. **(C)** Score of immune microenvironment between groups with high and low expression of GSDMD. **(D)** GSDMD high- and low-expression group TMB box plot. GSDMD, gasdermin D; TMB, tumor mutational burden. *(p<0.05), **(p<0.01), ***(p<0.001).

CIBERSORT immunological infiltration shows that “B cells naive”, “NK cells resting”, “Monocytes”, “Macrophages M1”, and “Mast cells activated” have a significant difference in the infiltration level of immune cells or immune gene sets between the two groups (**Figure 6A**), wherein “Monocytes”, “B cells naive”, and “Mast cells activated” have a significant correlation between the infiltration level and the expression level of GSDMD (**Figure 6B**).

The immune infiltration score shows that the “Stromal Score” and “Immune Score” have significant differences between the high- and low-expression groups of GSDMD. Among them, the low-expression group had a higher “Stromal Score”, while the high-expression group had a higher “Immune Score” (**Figure 6C**).

We calculated the TMB values of each sample based on the mutation data of clear cell renal cell carcinoma in TCGA database. Statistical analysis showed that there were significant differences in TMB values between the high- and low-expression groups of GSDMD, and there was a significant positive correlation between TMB and GSDMD expression levels (**Figure 6D**).

### 3.8 Immunohistochemical and clinical data analysis results from immunohistochemistry of GSDMD

**Figure 7:**
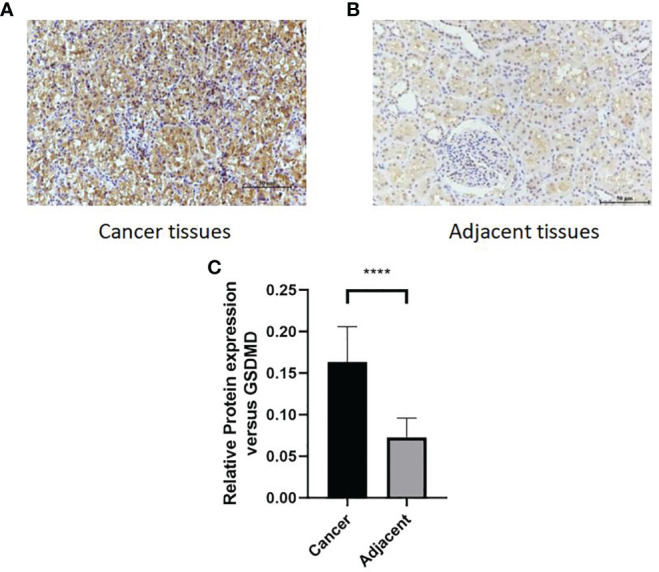
Comparison of protein expression of GSDMD in adjacent tissues and cancer tissues. **(A, B)** High staining in cancer tissues. Low staining in adjacent tissues. **(C)** The immunohistochemical staining results of 65 patients in two groups were statistically analyzed at p < 0.0001. GSDMD, gasdermin D. ****(p<0.0001).

After immunohistochemical staining of normal kidney tissues of 65 patients with cancer tissues (**Figure 7A**) and adjacent tissues (**Figure 7B**) in the First Affiliated Hospital of Xinjiang Medical University, it was found that the expression of GSDMD in cancer tissues was significantly higher than that in adjacent tissues (**Figure 7C**) (n = 65, p < 0.0001).

### 3.9 Correlation between GSDMD expression and clinical markers

As shown in **Table 2**, the expression of GSDMD immunohistochemical protein was correlated with TNM stage, Fuhrman grade, lymph node metastasis, sex, and smoking. However, it was not related to age, tumor location, tumor size, diabetes, hypertension, clinical symptoms, and serum cystatin C level.

**Table 2:** Correlation between GSDMD expression and clinical markers.

	Clinical variable	Number of cases	Average optical density, AOD (x̄ ± SD)	p
***Gender***	Male	39	0.1736 ± 0.04240	0.0156
	Female	26	0.1480 ± 0.03795	
***Age***	≤50 years	24	0.1641 ± 0.03878	0.9106
	>50 years	41	0.1629 ± 0.04470	
***Location***	Right	33	0.1597 ± 0.03850	0.4841
	Center	32	0.1671 ± 0.04622	
***TNM***	T1a–T2b	47	0.1540 ± 0.03535	0.0033
	T3a–T4	18	0.1877 ± 0.04986	
***Grade***	G1/G2	35	0.1519 ± 0.03570	0.0175
	G3/G4	30	0.1767 ± 0.04598	
***Size***	<7 cm	36	0.1596 ± 0.04278	0.4276
	≥7 cm	29	0.1680 ± 0.04197	
***Diabetes***	No	58	0.1637 ± 0.04249	0.8288
	Yes	7	0.1600 ± 0.04381	
***HBP***	No	36	0.1695 ± 0.05005	0.1902
	Yes	29	0.1556 ± 0.02912	
***Smoke***	No	33	0.1516 ± 0.03632	0.0218
	Yes	32	0.1754 ± 0.04511	
***Symptom***	No	39	0.1635 ± 0.03832	0.9611
	Yes	26	0.1630 ± 0.04847	
***Lymphatic Metastasis***	No	56	0.1586 ± 0.03877	0.0234
	Yes	9	0.1927 ± 0.05339	
***BMI***	<28	41	0.1679 ± 0.04373	0.2547
	>=28	24	0.1555 ± 0.03938	
***CysC***	Normal	51	0.1653 ± 0.04113	0.4896
	Abnormal (>1.2)	14	0.1563 ± 0.04730	

As shown, the expression of GSDMD immunohistochemical protein was correlated with three markers. However, it was not related to others.

GSDMD, gasdermin D; AOD, average optical density; HBP, high blood pressure; BMI, body mass index.

### 3.10 Correlation between expression of GSDMD and prognosis in patients with clear cell renal cell carcinoma

**Figure 8:**
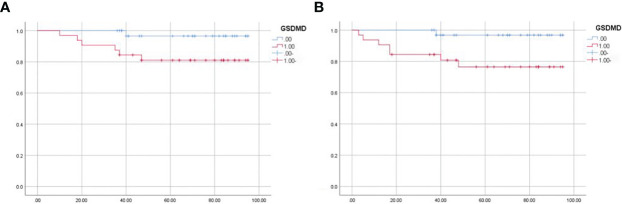
Survival analysis of 65 patients. **(A)** The overall survival of 65 patients analyzed by log-rank test (p = 0.048). **(B)** The disease-free survival of 65 patients analyzed by log-rank test (p = 0.021).

Sixty-five patients were divided into the high-expression group and low-expression group according to the median expression of GSDMD. The median follow-up time was 36 months. During the follow-up, eight patients metastasized or recurred, and seven patients died. The survival data were plotted by the K-M method and then analyzed by log-rank test. It was shown that the expression of GSDMD was correlated with OS (p = 0.048) (**Figure 8A**) and disease-free survival (DFS) (p = 0.021) (**Figure 8B**), and the prognostic survival time and disease-free survival time of patients with low expression were longer.

### 3.11 Expression of GSDMD in different cell lines

**Figure 9:**
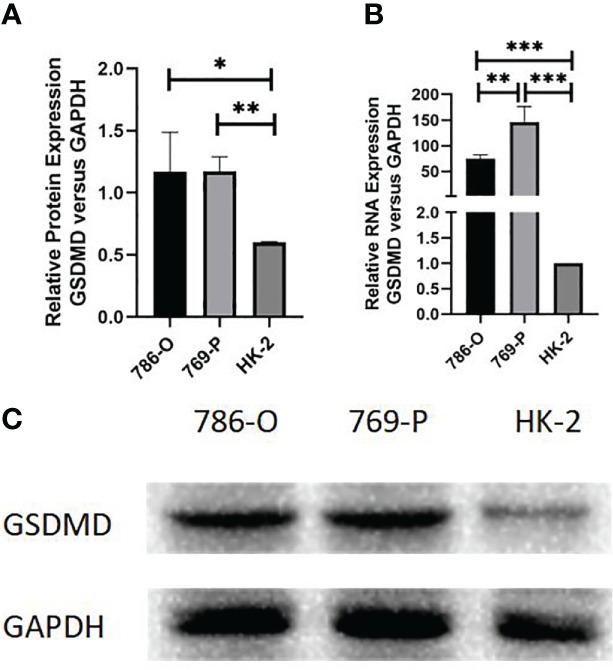
Expression of GSDMD in cell lines. **(A)** Detection of protein differences expression of GSDMD in three cell lines by Western blotting; 786-O cells (p = 0.036) and 769-P cells (p = 0.001) were statistically different from HK-2 cells. **(B)** Detection of mRNA differences expression of GSDMD in three cell lines by Western blotting; 786-O cells (p = 0.003) and 769-P cells (p = 0.015) were statistically different from HK-2 cells, and the mRNA expression of GSDMD in 786-O cells and 769-P cells was also statistically different (p = 0.018). This shows that 769-P cells are expressed more obviously. **(C)** Western blotting image of protein expression in three cell lines. GSDMD, gasdermin D. *(p<0.05), **(p<0.01), ***(p<0.001).

We further detected the mRNA level protein expression of GSDMD in 769-P, 786-O, and HK-2 cells by RT-PCR and Western blotting. The results showed that compared with normal renal cells, the protein expression level of the two kinds of renal cell carcinoma cells was significantly higher than that of normal renal cells (786-O *vs.* HK-2, p = 0.036; 769-P *vs.* HK-2, p = 0.001) (**Figures 9A, C**), and the mRNA expression level of GSDMD in the two kinds of renal cell carcinoma cells was also significantly higher than that of normal renal cells (786-O *vs.* HK-2, p = 0.003; 769-P *vs.* HK-2, p = 0.015; 769-P *vs.* 786-O, p = 0.018) (**Figure 9B**).

Because the difference between the 769-P cell line is more obvious than that of the 786-O cell line, the follow-up experiment was carried out on the basis of the 769-P cell line.

### 3.12 *In vitro* expression of DEGs in the high- and low-expression groups of GSDMD

**Figure 10:**
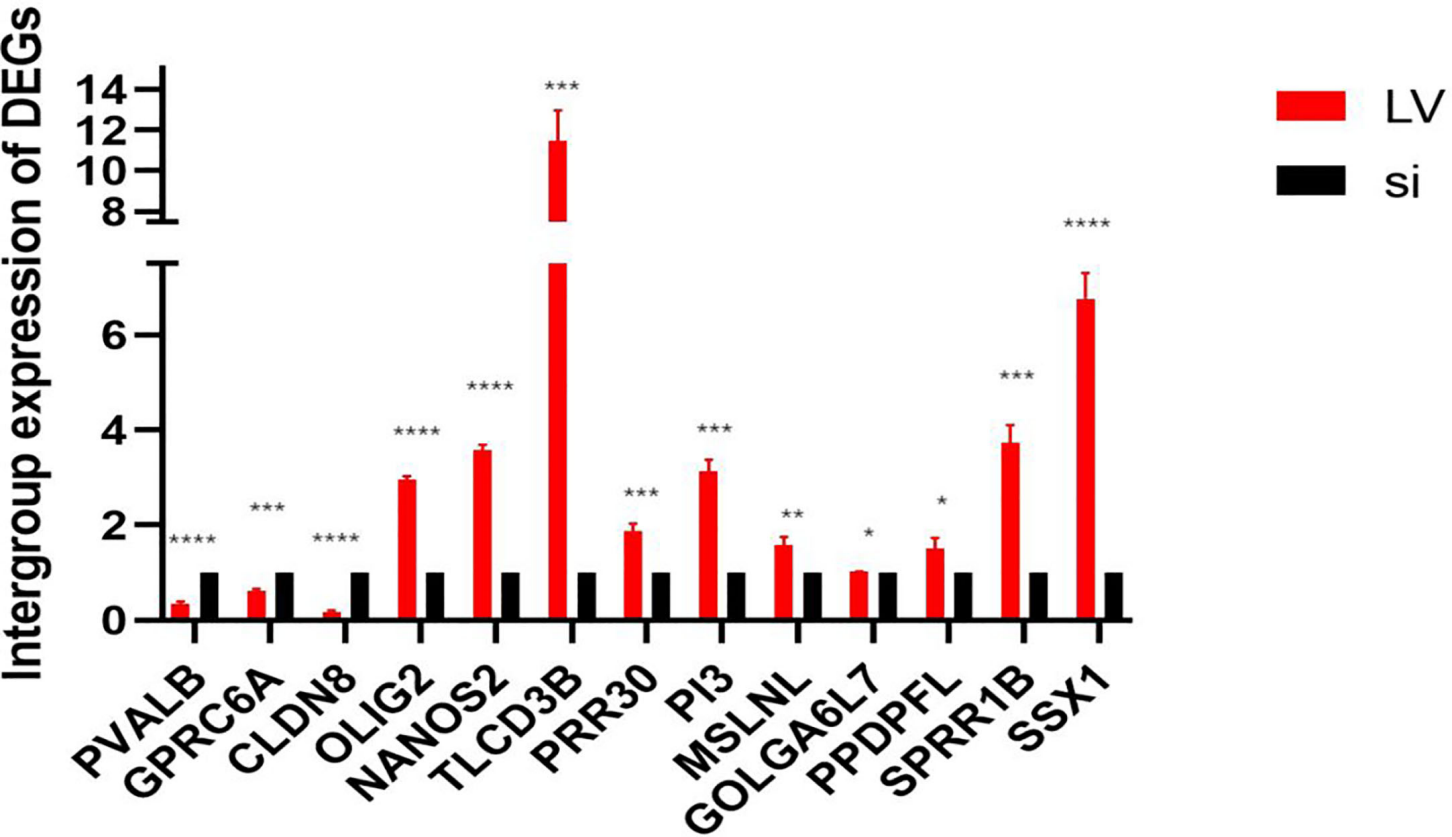
*In vitro* expression of DEGs in the high- and low-expression groups of GSDMD. This figure shows *in vitro* experimental data of differentially expressed genes in GSDMD. This picture shows three downregulated genes (PVALB (p < 0.0001), CLDN8 (p < 0.0001), and GPRC6A (p = 0.0002)) and 10 upregulated genes (PRR30 (p = 0.0007), GOLGA6L7 (p = 0.0251), TLCD3B (p = 0.0003), OLIG2 (p < 0.0001), PI3 (p = 0.0001), MSLNL (p = 0.005), NANOS2 (p < 0.0001), PPDPFL (p = 0.015), SPRR1B (p = 0.0002), and SSX1 (p < 0.0001)). DEGs, differentially expressed genes; GSDMD, gasdermin D. *(p<0.05), **(p<0.01), ***(p<0.001), ****(p<0.0001).

We used PCR to detect 13 differentially expressed genes (DEGs) in TCGA database between stable high-expression and low-expression 769-P cells constructed *in vitro*. The results showed that all 13 genes had expression differences *in vitro* cell lines, which were consistent with the data in TCGA database (**Figure 10**).

### 3.13 Effect of GSDMD on proliferation of renal cell carcinoma cells

**Figure 11:**
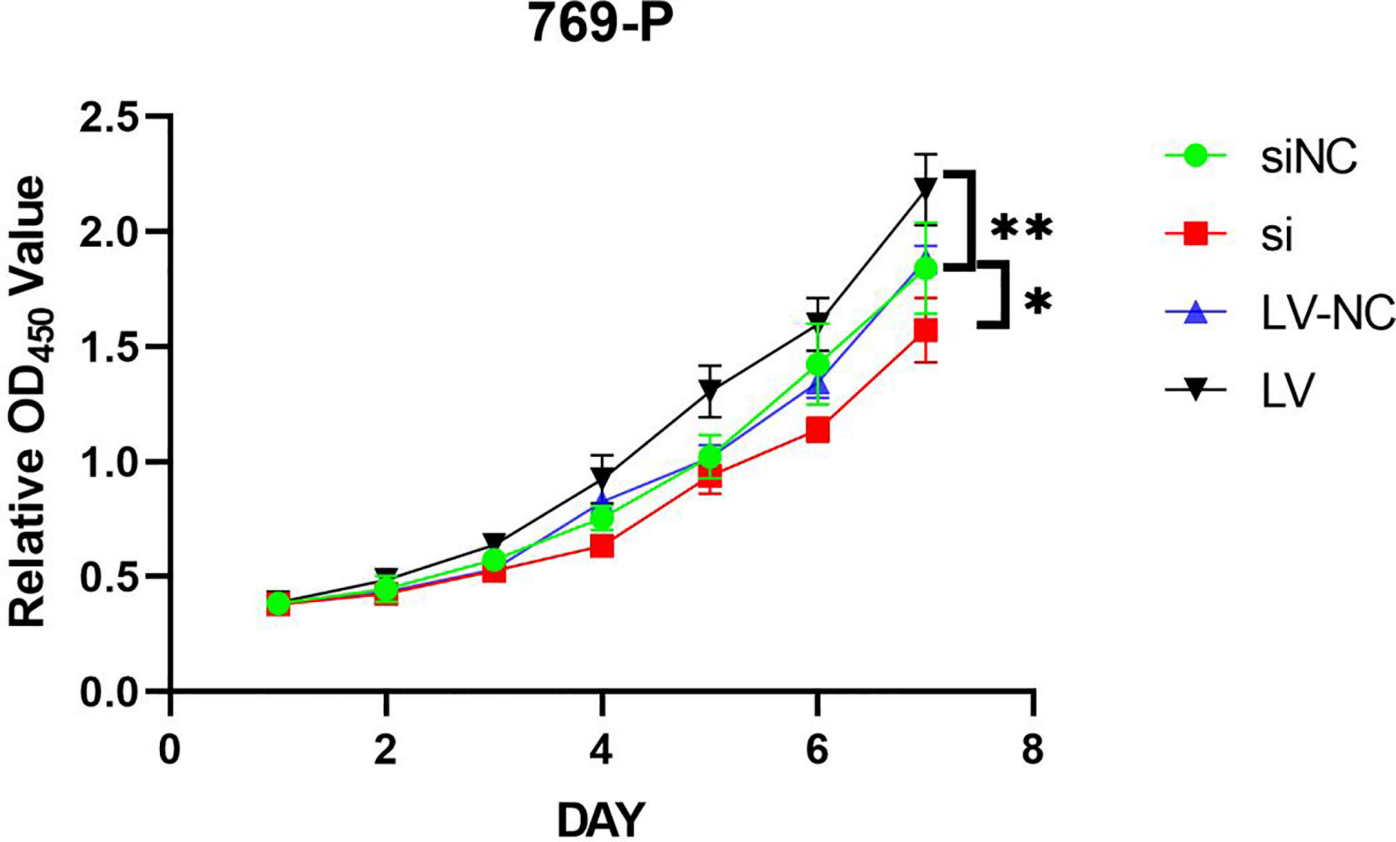
769-P transfected cell line proliferation was detected by MTT assays. In GSDMD upregulated group, the upregulated cells grew faster (p = 0.003); in GSDMD downregulation group, the growth of downregulated cells was slower (p = 0.022). GSDMD, gasdermin D. *(p<0.05), **(p<0.01).

MTT assay showed that the proliferation of 769-P-si-GSDMD was significantly lower than that of 769-P-si-NC-GSDMD (p = 0.022). Compared with 769-P-LV-NC-GSDMD, the proliferation ability of 769-P-LV-GSDMD cells was significantly increased (p = 0.003) (**Figure 11**).

### 3.14 Effects of GSDMD overexpression and knockdown on apoptosis of 769-P cells

**Figure 12:**
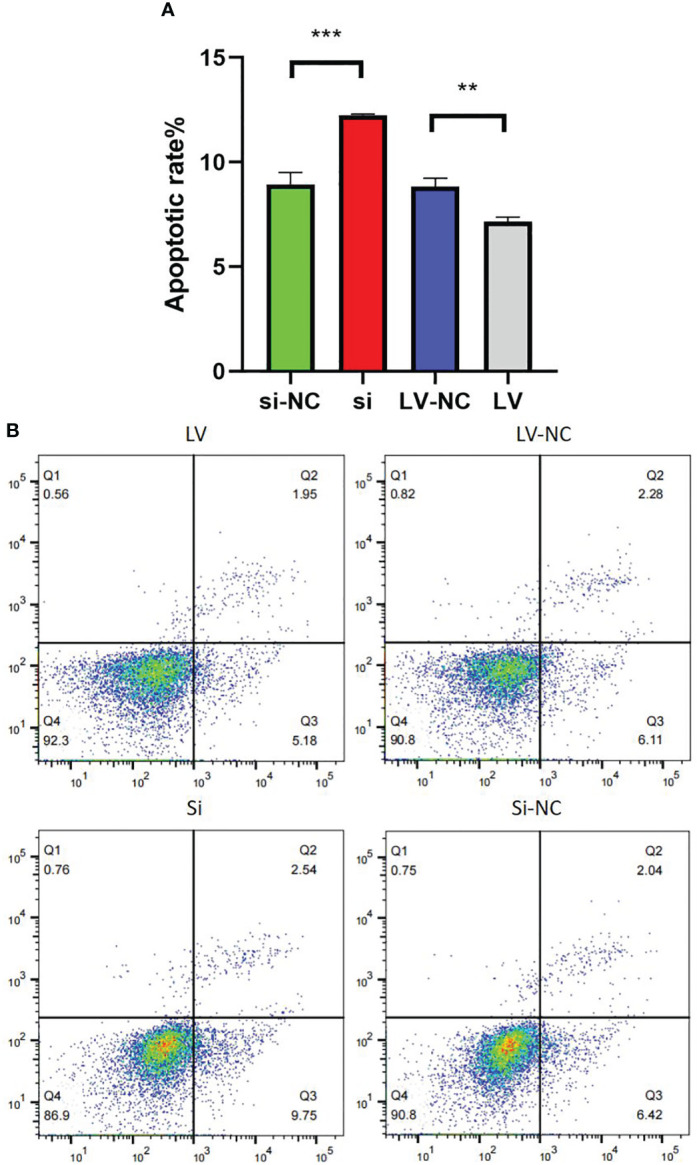
Detection of 769-P transfected cell line in apoptosis experiment. **(A)** In GSDMD upregulated group, the upregulated cells had a higher apoptosis rate (p = 0.003); in GSDMD downregulation group, the downregulated cells had a lower apoptosis rate (p = 0.001). **(B)** The flow cytometry picture of apoptosis experiment. GSDMD, gasdermin D. **(p<0.01), ***(p<0.001).

Compared with 769-P-si-NC-GSDMD, the apoptosis ability of 769-P-si-GSDMD increased (p = 0.001). Compared with 769-P-LV-NC-GSDMD, the apoptosis ability of 769-P-LV-GSDMD cells decreased (p = 0.003) (**Figures 12A, B**).

### 3.15 Effects of GSDMD overexpression and knockdown on invasion of 769-P cells

**Figure 13:**
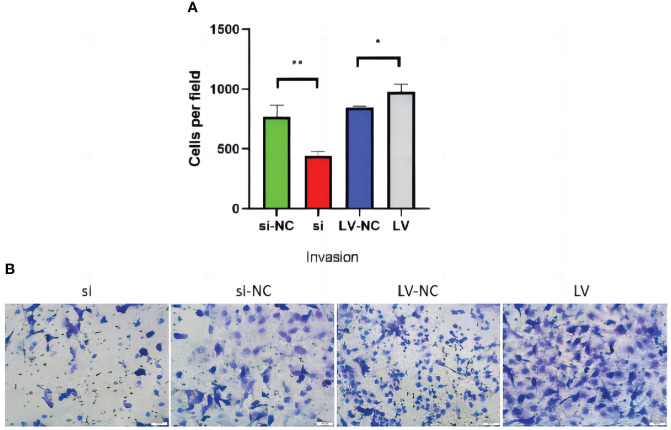
Transwell invasion of 769-P transfected cell lines. **(A)** In the invasion experiment, the number of cells passing through the room of cell lines with GSDMD upregulated increased (p = 0.01), while the number of cells downregulated decreased (p = 0.005). **(B)** The Transwell picture of cell invasion test. GSDMD, gasdermin D.

Transwell invasion assay showed that si decreased the ability of invasion compared with si-NC (p = 0.01). Compared with LV-NC, the invasion ability of LV cells was significantly increased (p = 0.005) (**Figures 13A, B**).

### 3.16 Effects of GSDMD overexpression and knockdown on migration of 769-P cells

**Figure 14:**
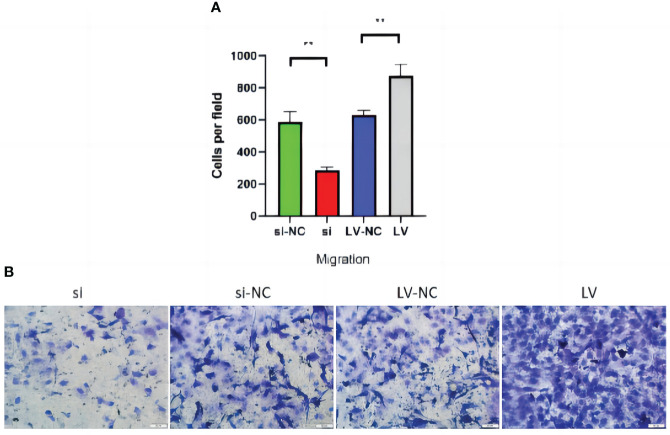
Transwell migration of 769-P transfected cell lines. **(A)** In the migration experiment, the number of cells passing through the room of cell lines with GSDMD upregulated increased (p = 0.006), while the number of cells downregulated decreased (p = 0.001). **(B)** The Transwell picture of cell migration test. GSDMD, gasdermin D.

Transwell migration assay showed that si decreased the ability of migration compared with si-NC (p = 0.006). Compared with LV-NC, the migration ability of LV cells was significantly increased (p = 0.001) (**Figures 14A, B**).

## 4 Discussion

Worldwide, RCC is the seventh most common cancer in men and eighth in women, accounting for 5% and 3% (10) of all tumor diagnoses, respectively. In recent years, drug therapy for renal cell carcinoma has developed rapidly, and many therapeutic methods such as targeted therapy and immunotherapy have emerged. Although it improves the living environment of patients with refractory renal cell carcinoma, their respective shortcomings are still obvious, including low effective rate and drug resistance (11–14). Therefore, finding new diagnostic and therapeutic targets is the focus of renal cell carcinoma treatment in the future.

Pyroptosis is a new programmed cell death mediated by inflammation in recent years. Caspase-1/4/5/11 is activated by inflammatory corpuscles or lipopolysaccharide (LPS), and then the common substrates GSDMD are cut into N-terminal (GSDMD-N) and C-terminal (GSDMD-C). Activated GSDMD-N punched holes in the cell membrane to form channels, which destroyed the balance of ions on both sides of the cell membrane, and finally led to cell swelling, lysis, and final death (15, 16). In 2015, GSDMD was identified as the key molecule for the formation of plasma membrane pores in pyroptosis for the first time (17).

In different cancers, GSDMD plays different roles in the occurrence and development of tumors. On the one hand, pyroptosis can inhibit the growth of tumors by promoting cell death. On the other hand, the essence of pyroptosis is an inflammatory reaction, which can provide a suitable environment for the growth of tumor cells, thus promoting the growth of tumor (18). For example, in gastric cancer, the expression of GSDMD is reduced, which promotes the growth and proliferation of tumors (5). The expression of GSDMD increased in non-small cell lung cancer and metastatic melanoma, which increased the invasion and apoptosis of tumors (6). It has been found that there is an interaction between pyroptosis and apoptosis. In the absence of GSDMD, Caspase-1 can activate Caspase-3/7 and induce apoptosis. In the process of apoptosis, Caspase-3/7 can cut GSDMD at the Asp87 site to make it lose its activity and inhibit pyroptosis (19, 20). Therefore, what role does GSDMD play in kidney cancer?

To explore the role of GSDMD in the development of renal cell carcinoma and the correlation between GSDMD and the stage and prognosis of renal cell carcinoma patients, we did the above experiments and obtained the following results. First, through the biological information platform database, the expression of GSDMD in renal cell carcinoma and normal renal tissue and its relationship with clinical stage and prognosis were analyzed. The results showed that the expression of GSDMD in renal cell carcinoma was higher than that in normal renal tissue, and there were differences in the clinical stage and pathological stage. Subsequently, we found that GSDMD also has certain significance in the diagnosis of renal cell carcinoma through the ROC curve. Next, we used the GEO database to analyze the expression of GSDMD in 72 cases of clear cell renal cell carcinoma and 72 normal patient tissues and plotted ROC curves to validate the results of TCGA database. In addition, we verified that the expression of GSDMD in renal cell carcinoma was significantly higher than that in normal kidney tissue by immunohistochemistry and cell experiments *in vitro*, which had statistical differences. The data of 65 patients with ccRCC in the First Affiliated Hospital of Xinjiang Medical University were analyzed, and the expression of GSDMD was correlated with TNM stage, Fuhrman grade, lymph node metastasis, sex, and smoking (p < 0.05). However, there was no significant correlation between GSDMD and age, tumor location, tumor size, diabetes, hypertension, clinical symptoms, alkaline phosphatase level, serum cystatin C level, and venous tumor thrombus (p > 0.05). Meanwhile, the expression of GSDMD was correlated with disease-free survival and overall survival of renal cell carcinoma (p < 0.05). All the above experiments have been verified in previous bioinformatics analyses, suggesting that GSDMD is a potential biomarker with diagnosis and prognosis significance in ccRCC.

Many studies have confirmed that the occurrence, progress, or recurrence of tumors will be affected by the infiltration of immune cells in the tumor microenvironment, which may be an important factor in the efficacy and clinical results of immunotherapy (21), and tumor-infiltrating lymphocyte (TILs) in the tumor microenvironment (TME) are independent predictors of the prognosis of many cancers and the efficacy of immunotherapy (22). Due to the high mutational burden (TMB) of tumors, a large amount of antigens can be produced in the body, which can enable patients to have a better response to immunotherapy. In order to explore the possible role of GSDMD in the treatment of tumors, we found that the content of stromal cells decreased but the content of immune cells increased in the high-expression group through immune microenvironment analysis. Further research on the expression of immune cells between the two groups found that in “B cells naive”, “NK cells resting”, “Monocells”, and “Macrophages M1”, there is a significant difference in the infiltration level of immune cells or immune gene sets such as “Mast cells activated” between the two groups. The infiltration levels of “Monocytes”, “B cells naive”, and “Mast cells activated” are significantly correlated with the expression level of GSDMD. At the same time, there was a significant statistical difference in the mutation load of TMB response between the high- and low-expression groups, and the expression level of GSDMD was significantly positively correlated with TMB. All the above indications indicate that GSDMD may serve as an indicator for measuring the treatment effectiveness of clear cell renal cell carcinoma patients.

In order to further study the function of GSDMD, we screened 507 differentially expressed genes between the high- and low-expression groups of GSDMD through TCGA database. We screened 24 genes related to GSDMD expression through GeneMANIA and STRING database, constructed PPI based on GSDMD, and analyzed GO and KEGG enrichment. The results showed that these genes play a role in the processes and pathways of pyroptosis, apoptosis, necrosis, inflammatory reaction, cytokine secretion, NIK/NF-κB signaling, NOD-like receptor signaling pathway, cytoplasmic DNA sensing pathway, antigen processing and presentation, etc. At present, studies have shown that NF-κB can regulate tumor angiogenesis and invasiveness (23). Moreover, NOD-like receptors, as a very significant link in innate immune response, can also promote apoptosis of tumor cells (24). It is found that the NOD1 receptor can inhibit tumor development after being inhibited (25), which indicates that the NOD-like receptor pathway also plays a dual role in promoting and inhibiting tumor occurrence and development.

In order to further investigate the strongest and statistically significant correlation between the high- and low-expression groups of GSDMD, we constructed stable cell lines with high expression and knockdown GSDMD of LV/LV-NC and si/si-NC and validated the expression of 13 genes. The results confirmed that the differential expression of 13 genes was statistically significant, which is consistent with the results of TCGA database. Many studies have found that multiple genes play a crucial role in regulating the biological behavior of tumors. Among the downregulated genes, PVALB passes through AKT/GSK-3β pathways that inhibit cell growth and induce cell death (26), and CLDN8 inhibits the proliferation, migration, and invasion of 786-O ccRCC cells through the EMT and AKT pathways (27). Among the upregulated genes, in the MYC amplified MB PDX model, tumors with low OLIG2 expression are radiation sensitive and have fewer relapses, while tumors with high OLIG2 expression have high radiation resistance and a significantly increased probability of recurrence (28), a predictive signature based on the expression of PI3 could be an independent prognostic factor for ccRCC (29). NANOS2 inhibits rheb by inhibiting transcription to inhibit mTORC1 activity, thereby inhibiting the cell cycle (30); SPRR1B is upregulated by the proinflammatory cytokines IL-1beta and IFN-gamma *via* p38 MAPK-mediated signaling pathways that lead to the activation of transcription factors CREB and ZEB1, respectively. These results identify key intracellular signaling intermediates involved in the pathogenesis of immune-mediated ocular surface squamous metaplasia (31). The main function of SSX1 is to form fusion genes with multiple genes, thereby demonstrating its impact on the biological function of tumor cells. For example, the deletion of SS18-SSX1 inhibits the activity of synovial sarcoma and induces cell apoptosis (32).

Studies have shown that in the process of tumor occurrence and development, tumor cells derived from epithelial cells will acquire the abilities of movement and invasion after EMT and then metastasize to the distal end through blood and lymphatic circulation, and metastasis is the main cause of death of malignant tumors (33). It is known that there are many signals that can trigger the EMT of tumor cells, including hypoxia, miRNA, and transforming growth factor (34). We studied the correlation between GSDMD- and EMT-related molecules in ccRCC. We found that GSDMD was significantly correlated with the expression of many genes in EMT-related molecules. The differential expression of GSDMD may play an important role in the metastasis and invasion of clear cell renal cell carcinoma.

Finally, in order to prove the role of GSDMD in the occurrence and development of renal cell carcinoma, we utilized stable cell lines of LV/LV-NC and si/si-NC and carried out *in vitro* functional experiments such as MTT proliferation assay, Transwell invasion and migration assay, and apoptosis assay. The results showed that overexpression of GSDMD promoted the proliferation, invasion, and migration of renal cell carcinoma but decreased the apoptosis ability of renal cell carcinoma. Knocking down GSDMD inhibited the proliferation, invasion, and migration of renal cell carcinoma and increased the apoptosis of renal cell carcinoma, which also confirmed the results of the above bioassay.

Although this study investigated the possible role of GSDMD in the occurrence, development, and treatment of clear cell renal cell carcinoma through patient tissue experiments and *in vitro* experiments, there are still many shortcomings, such as the specific pathway through which GSDMD plays these roles remains to be studied.

In conclusion, GSDMD exists as an oncogene in the occurrence and development of renal cell carcinoma and is related to the prognosis of ccRCC, and its expression increases with the increase of stage and grade, which may become a new marker and therapeutic target in the future treatment of renal cell carcinoma.

## Data availability statement

The original contributions presented in the study are included in the article/**Supplementary Material**. Further inquiries can be directed to the corresponding authors.

## Ethics statement

The studies involving human participants were reviewed and approved by Medical Ethics Committee of the First Affiliated Hospital of Xinjiang Medical University. Written informed consent for participation was not required for this study in accordance with the national legislation and the institutional requirements.

## Author contributions

JZ, AA, YW, and JM contributed to conception and design of the study. JZ organized the database. JZ performed the statistical analysis. JZ wrote the first draft of the manuscript. AA, YW, and JM wrote sections of the manuscript. All authors contributed to article and approved the submitted version.
